# Structural, thermodynamic, and phosphatidylinositol 3‐phosphate binding properties of Phafin2

**DOI:** 10.1002/pro.3128

**Published:** 2017-02-13

**Authors:** Tuo‐Xian Tang, Ami Jo, Jingren Deng, Jeffrey F. Ellena, Iulia M. Lazar, Richey M. Davis, Daniel G. S. Capelluto

**Affiliations:** ^1^ Protein Signaling Domains Laboratory, Department of Biological SciencesBiocomplexity Institute, and Center for Soft Matter and Biological Physics, Virginia TechBlacksburgVirginia24061; ^2^ Department of Chemical EngineeringVirginia TechBlacksburgVirginia24061; ^3^ Department of Biological SciencesVirginia TechBlacksburgVirginia24061; ^4^ Biomolecular Magnetic Resonance Facility, University of VirginiaCharlottesvilleVirginia22904

**Keywords:** Phafin2, phosphatidylinositol 3‐phosphate, conformation, protein structure

## Abstract

Phafin2 is a phosphatidylinositol 3‐phosphate (PtdIns(3)P) binding protein involved in the regulation of endosomal cargo trafficking and lysosomal induction of autophagy. Binding of Phafin2 to PtdIns(3)P is mediated by both its PH and FYVE domains. However, there are no studies on the structural basis, conformational stability, and lipid interactions of Phafin2 to better understand how this protein participates in signaling at the surface of endomembrane compartments. Here, we show that human Phafin2 is a moderately elongated monomer of ∼28 kDa with an intensity‐average hydrodynamic diameter of ∼7 nm. Circular dichroism (CD) analysis indicates that Phafin2 exhibits an α/β structure and predicts ∼40% random coil content in the protein. Heteronuclear NMR data indicates that a unique conformation of Phafin2 is present in solution and dispersion of resonances suggests that the protein exhibits random coiled regions, in agreement with the CD data. Phafin2 is stable, displaying a melting temperature of 48.4°C. The folding‐unfolding curves, obtained using urea‐ and guanidine hydrochloride‐mediated denaturation, indicate that Phafin2 undergoes a two‐state native‐to‐denatured transition. Analysis of these transitions shows that the free energy change for urea‐ and guanidine hydrochloride‐induced Phafin2 denaturation in water is ∼4 kcal mol^−1^. PtdIns(3)P binding to Phafin2 occurs with high affinity, triggering minor conformational changes in the protein. Taken together, these studies represent a platform for establishing the structural basis of Phafin2 molecular interactions and the role of the two potentially redundant PtdIns(3)P‐binding domains of the protein in endomembrane compartments.

**Abbreviations**
AUC: analytical ultracentrifugation
CD: circular dichroism
DLS: dynamic light scattering
*ƒ/ƒ_0_*: frictional ratio
GuHCl: guanidine hydrochloride
PtdIns(3)P: phosphatidylinositol 3‐phosphate
*s*: sedimentation coefficient
*S*_20,w_: sedimentation coefficient under standard conditions
SPR: surface plasmon resonance

## Introduction

**Figure 1:**
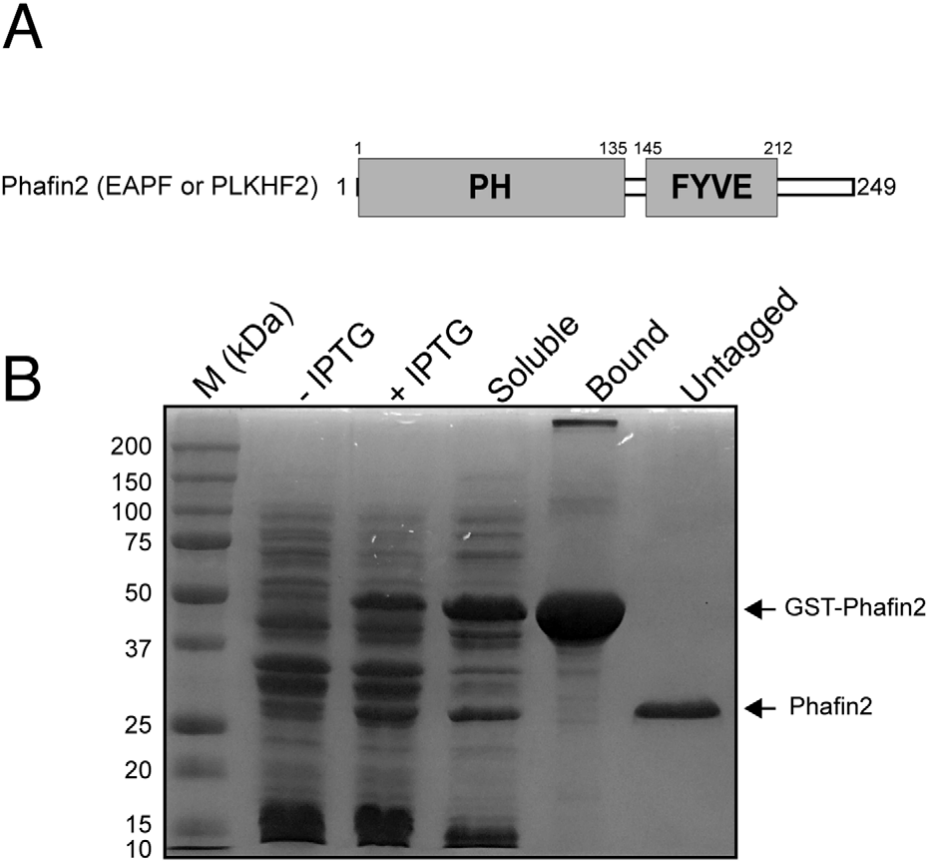
Purification of human Phafin2. A: Modular organization of Phafin2. B: SDS‐PAGE showing IPTG‐induced overexpression and purification of GST‐Phafin2.

Phafin2 (also known as EAPF or PLEKHF2) belongs to the Phafin family of proteins containing an N‐terminal PH (Pleckstrin homology) and C‐terminal FYVE (Fab1, YOTB, Vac1, and EEA1) domains [Fig. 1(A)].^1^ Both PH and FYVE domains of Phafin2 are phosphatidylinositol 3‐phosphate (PtdIns(3)P) binding modules.^2^ PtdIns(3)P is a hallmark of endosomes as it mediates the recruitment of effector proteins to these compartments; thus, both PtdIns(3)P‐interacting domains can target Phafin2 to PtdIns(3)P‐enriched membranes. Phafin2 regulates the function and structure of endosomes through a Rab5‐dependent process^3^ although no direct interaction of Phafin2 and Rab5 occurs.^4^ Several lines of evidence suggest that Phafin2, the Phafin family member protein Phafin1, and the *Drosophila* homologue Rush promote the enlargement of early endosomes^3^, ^4^, ^5^, ^6^ with their FYVE domains playing a dominant role.^3^ Phafin2 interacts with the FYVE domain‐containing protein EEA1, which regulates endosomal cargo trafficking and fusion events, but does not directly participate in cargo sorting.^4^ A role for lysosomal Phafin2 in the induction of autophagy has also been recently reported. Phafin2‐mediated autophagy requires the presence of PtdIns(3)P at the lysosomal surface and the serine/threonine kinase Akt.^2^ Binding of Phafin2 to Akt requires both the PH and FYVE domains. The interaction of Phafin2 with PtdIns(3)P allows localization of the Phafin2‐Akt complex to the lysosomal surface.^2^ Phafin2 has previously been associated with caspase 12‐dependent apoptosis through the endoplasmic reticulum (ER)‐mitochondrial apoptotic pathway.^7^ In this pathway, Phafin2 translocates to the ER after tumor necrosis factor‐α (TNF‐α) stimulation, inducing apoptosis in a PH‐ and FYVE domain‐dependent manner.^7^ The action of Phafin2 favors a more rapid increase of the cytosolic Ca^2+^ levels, which enhances TNF‐α‐mediated apoptosis and suppresses the unfolding protein response at the ER.^7^

In this article, we report the first structural and thermodynamic characterization of Phafin2. We show that Phafin2 is a nonglobular monomer that contains α‐helical and β‐sheet elements. Temperature‐ and chemical‐induced denaturation studies indicate that Phafin2 transitions from native to denatured states, without the presence of long‐lived intermediate states. Binding of Phafin2 to PtdIns(3)P occurs with nanomolar affinity, which is accompanied by local conformational changes in the protein. PtdIns(3)P‐binding sites in Phafin2 are predicted to be located at conserved regions found in related PH and FYVE domains.

## Results and Discussion

### Phafin2 is an elongated monomer

**Figure 2:**
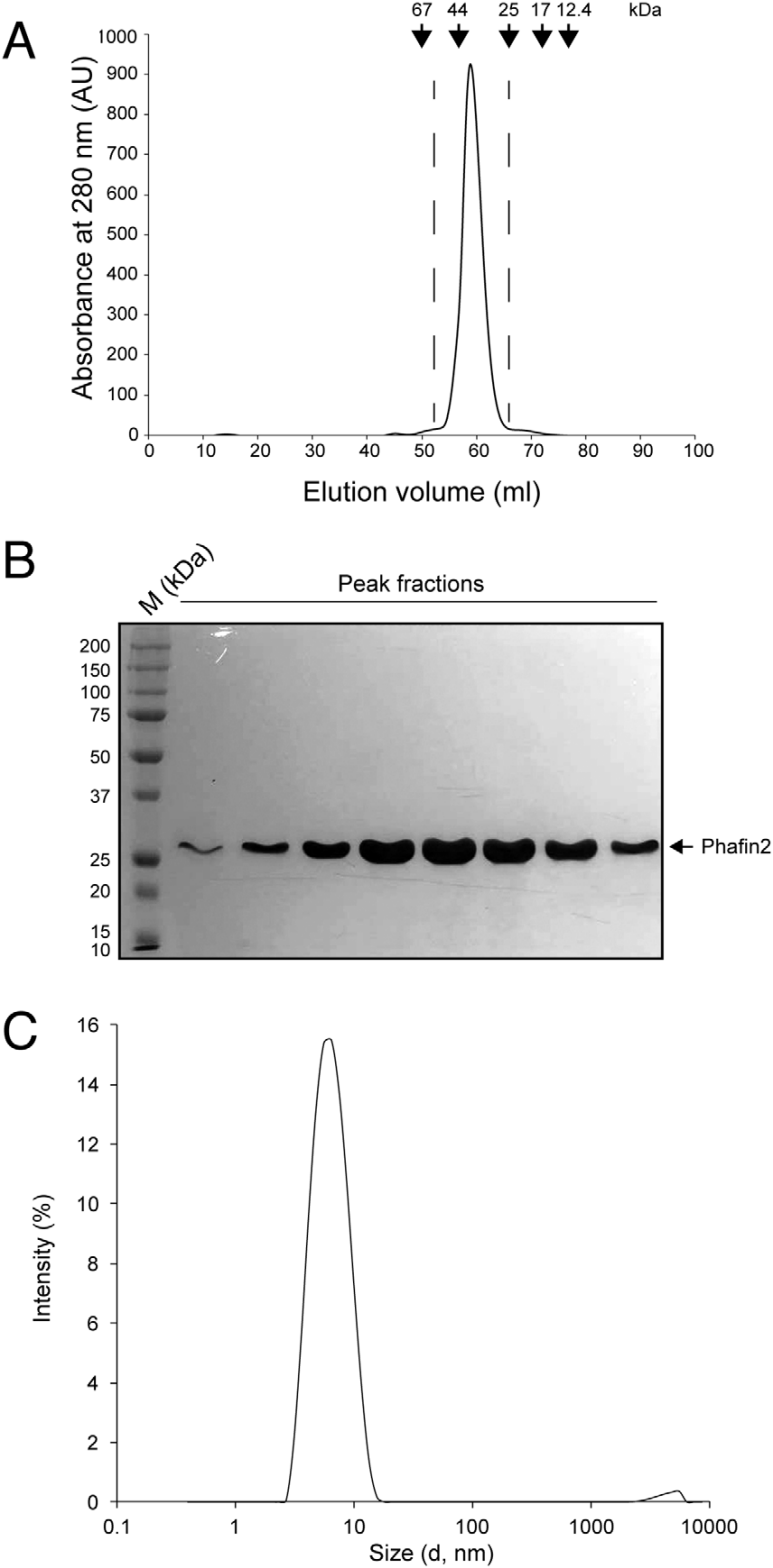
Phafin2 is an elongated monomer. A: Analytical size‐exclusion chromatography showing that Phafin2 eluted between ovalbumin and chymotrypsinogen A. The standards were: BSA (67 kDa), ovalbumin (44 kDa), chymotrypsinogen (25 kDa), myoglobin (17 kDa), and cytochrome C (12.4 kDa). B: SDS‐PAGE showing the purity and size of Phafin2 from the chromatographic peak displayed in (A). C: DLS plot of Phafin2 at 25°C.

**Figure 3:**
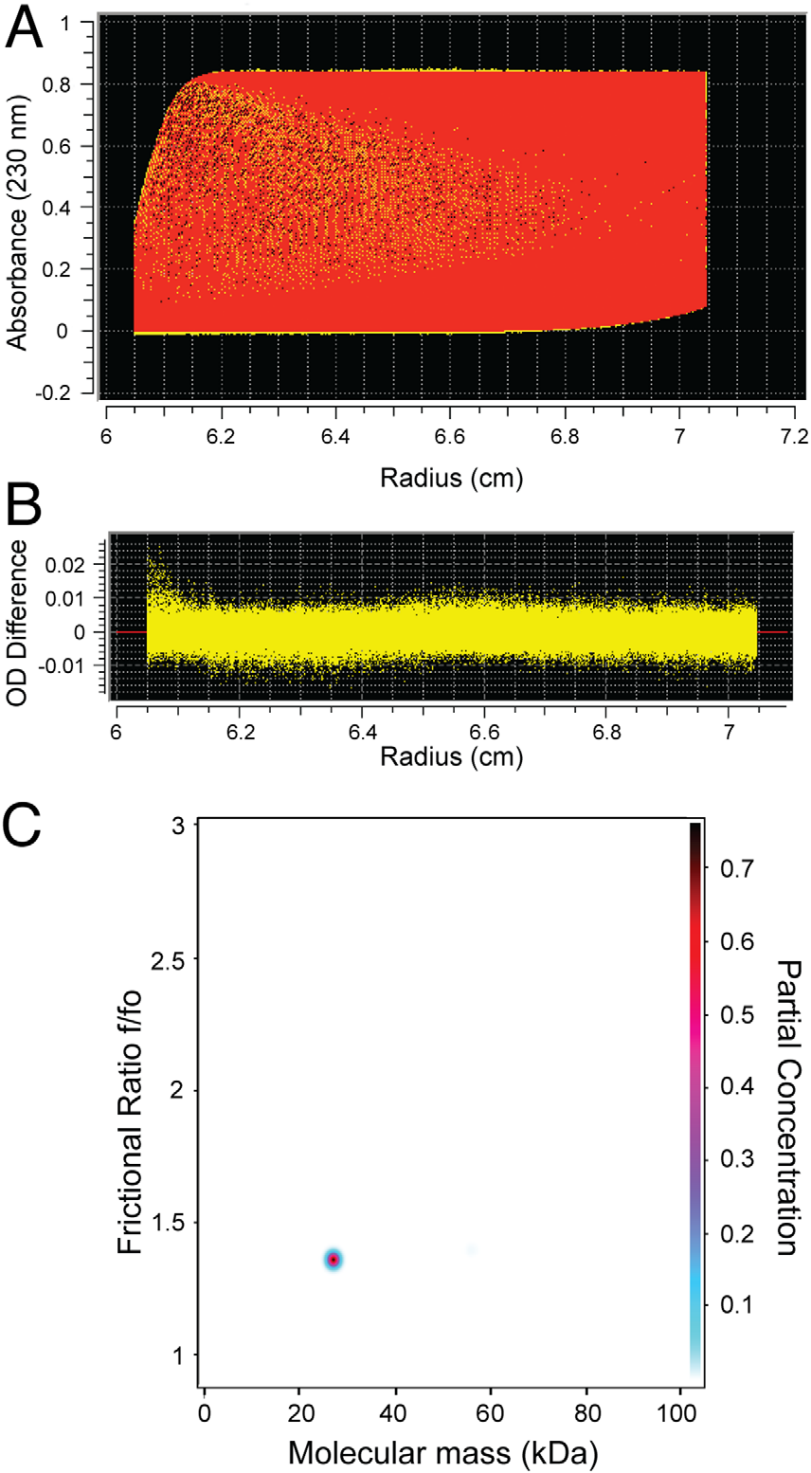
Sedimentation velocity AUC analysis of Phafin2. A: Representative interference scans of Phafin2 tracked during AUC. B: Representative residuals obtained from the data fitting analysis described in Methods. C: Representative molecular mass distribution of Phafin2 in relationship with its frictional ratio and partial concentration of the protein.

Optimal overexpression of glutathione S‐transferase (GST)‐tagged Phafin2 was obtained in the presence of Zn^2+^, as the protein presents a C‐terminal Zn^2+^ finger FYVE domain [Fig. 1(A)]. Soluble GST‐Phafin2 was immobilized on glutathione beads and the GST was removed by thrombin digestion. Recombinant Phafin2 remained soluble after removal of the GST tag. SDS‐PAGE analysis showed a protein band with a molecular mass of ∼28 kDa [Fig. 1(B)], close to the theoretical molecular mass of Phafin2 (27,941 Da). Removal of protein aggregates and other minor protein contaminants was carried out using an FPLC‐driven Superdex 75 size‐exclusion column (data not shown). MALDI‐TOF was used to identify Phafin2, given the proximity of its molecular mass to GST's molecular mass. Tryptic products of Phafin2 accounted for ∼77% amino acid sequence coverage of the protein, including its C‐terminus (Table 1, Supporting Information 1). To address whether the Phafin2 N‐terminus was also intact, the first five residues from the N‐terminus were also sequenced, identifying Gly‐Ser‐Met‐Val‐Asp, with the first two corresponding to the amino acids translated from the vector. Size‐exclusion chromatography was employed to study the hydrodynamic properties of Phafin2. Phafin2 consistently eluted as a single sharp peak with an estimated apparent molecular mass of ∼42 kDa, a value far from its theoretical molecular mass [Fig. 2(A,B)], suggesting that the protein exhibits an elongated structure. Dynamic light scattering (DLS) analysis indicated that more than 99% of the mass of Phafin2 was in a peak with an intensity‐average value of ∼7 nm at 25°C [Fig. 2(C)]. This data confirms that Phafin2 exhibits only one conformational state. Sedimentation velocity analytical ultracentrifugation (AUC) analysis showed that Phafin2 was monodispersed with an estimated molecular mass of 27,115 Da (Fig. 3), in close agreement with the predicted value for a monomeric state. Phafin2 displayed a sedimentation coefficient under standard conditions ( $S_{20,w}^{0}$) of 2.42 S and a frictional ratio (*f*/*f*_o_) of 1.35 [Fig. 3(C)]. The *f*/*f*_o_ ratio represents the frictional coefficient of an unknown molecule to that of an ideal spherical molecule of the same density and volume, which consequently, features the anisotropy of a molecule.^8^ The *f*/*f*_o_ value of Phafin2, which is over 1, indicates that the protein is asymmetrical with a moderately elongated structure.^9^

**Table 1:** Identification of Human Phafin2

Peptide	Phafin2	Sequence
1	14–34	RISIVENCFGAAGQPLTIPGR
2	15–44	ISIVENCFGAAGQPLTIPGRVLIGEGVLTK
3	35–47	VLIGEGVLTKLCR
4	74–97	YNKQHIIPLENVTIDSIKDEGDLR
5	98–107	NGWLIKTPTK
6	108–119	SFAVYAATATEK
7	120–128	SEWMNHINK
8	137–163	SGKTPSNEHAAVWVPDSEATVCMRCQK
9	140–165	TPSNEHAAVWVPDSEATVCMRCQKAK
10	177–191	KCGFVVCGPCSEKR
11	191–202	RFLLPSQSSKPVR
12	192–223	FLLPSQSSKPVRICDFCYDLLSAGDMATCQPAR
13	224–249	SDSYSQSLKSPLNDMSDDDDDDDSSD

MS/MS results obtained on peptides generated by trypsin proteolysis of Phafin2.

### Structural features of Phafin2

**Figure 4:**
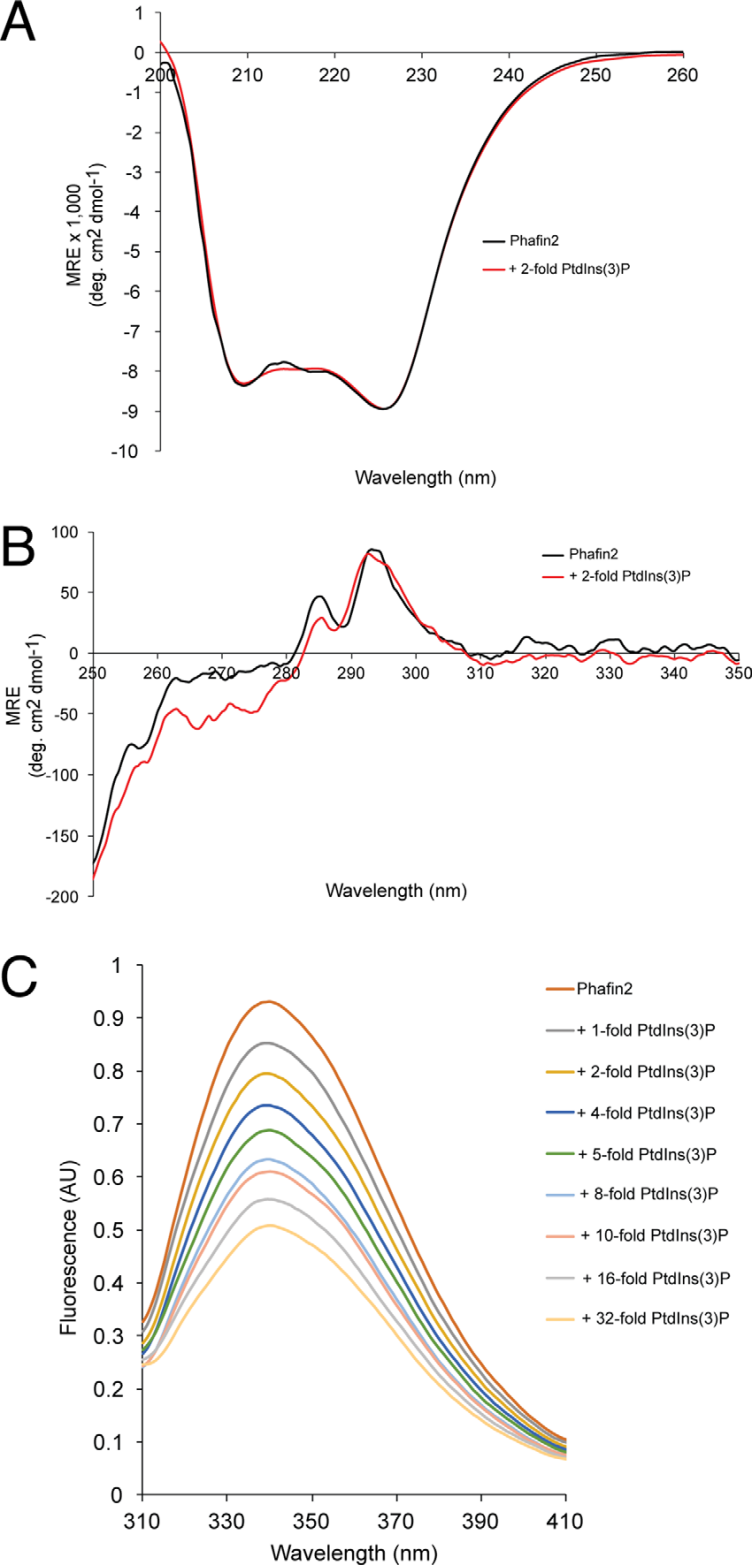
Phafin2 is an α/β protein that undergoes local conformational changes upon PtdIns(3)P binding. A: Far‐UV CD spectra of Phafin2 in the absence and presence of 2‐fold PtdIns(3)P. B: Near‐UV CD spectra of Phafin2 in the absence and presence of 2‐fold PtdIns(3)P. C: Intrinsic tryptophan fluorescence of Phafin2 titrated with PtdIns(3)P.

Far‐UV circular dichroism (CD) spectroscopy was carried out to study the secondary structural content of Phafin2. The spectrum of Phafin2 exhibited two minima at 208 and 226 nm [Fig. 4(A)], with the latter being more pronounced, suggesting that it is an α/β protein. By using the CDSSTR algorithm, the secondary structural content of Phafin2 was estimated to be 23% α‐helix, 21% β‐strand, 15% β‐turn, and 41% random coil (NRMSD = 0.018). The ratio of mean residue ellipticity (MRE) values at 222 and 208 nm can be used to characterize α‐helices in proteins. A ratio of ∼1.0 predicts that α‐helices exhibit extensive interhelical contact in helical bundles and coiled coil regions, whereas a ratio of ∼0.8 predicts limited interhelical contacts.^10^ The MRE values at 208 and 222 nm were −7,882.22 and −8,376.92 deg cm^2^ dmol^−1^, respectively, giving a ratio of 1.06. Thus, far‐UV CD analysis suggests that there are extensive interhelical contacts in Phafin2. Addition of the water‐soluble phosphoinositide ligand, dibutanoyl PtdIns(3)P, did not induce secondary structural changes in the protein [Fig. 4(A)]. The near‐UV CD spectrum provides valuable information about the tertiary structure of a protein.^11^ Phafin2 exhibits a spectrum dominated by the contributions of two positive bands from Trp residues, one negative band at 275 nm for Tyr and vibronic bands at 258 and 270 nm, which are often attributed to a region associated with Phe residues [Fig. 4(B)] (Phafin2 has three Trp, five Tyr, and nine Phe residues). Addition of water‐soluble dibutanoyl PtdIns(3)P led to minor changes in the region comprising the aromatic regions, with the exception of the positive band at 294 nm, a peak likely associated with Trp residues. Local conformational changes around one or more Trp residues in Phafin2 were also observed when the protein was titrated with water‐soluble dibutanoyl PtdIns(3)P. Addition of PtdIns(3)P decreased the intrinsic tryptophan fluorescence of the protein at 339 nm [Fig. 4(C)], suggesting that binding of the phosphoinositide changes the polarity of the environment near one or more Phafin2 Trp residues.

### Thermostability of Phafin2

**Figure 5:**
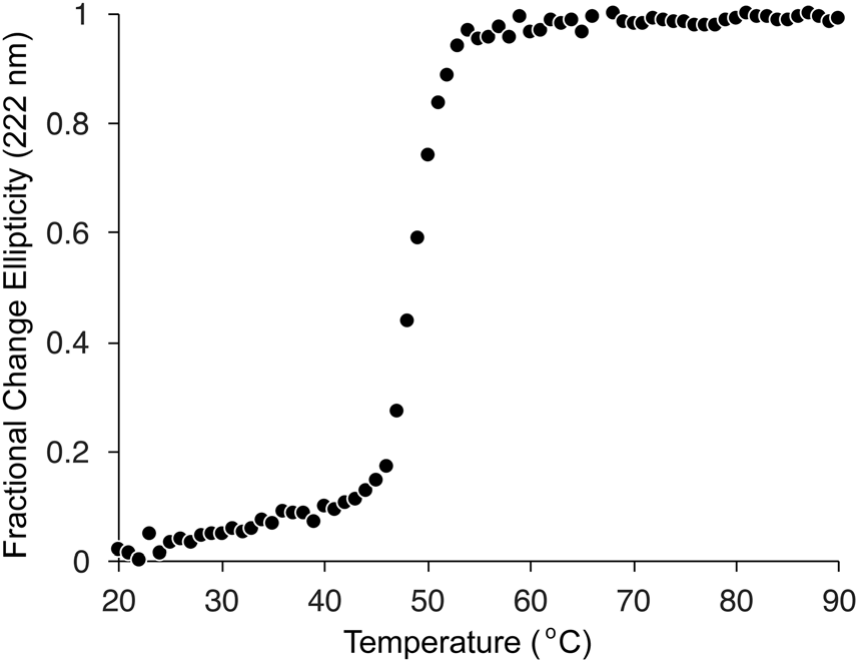
Thermostability of Phafin2. Thermal denaturation plot of Phafin2 monitored by CD spectroscopy. Data represents an average of three independent experiments.

Far‐UV CD at 222 nm was employed to monitor Phafin2's thermal stability and to estimate its melting temperature (*T*_M_), that is, the temperature at which half of the protein is unfolded. Phafin2 denatured with a single transition over the range of 20 to 90°C (Fig. 5). The *T*_M_ for Phafin2 is 48.4 ± 0.6°C. Significant precipitation was observed at the end of the denaturation curve, indicating that the transition was irreversible.

### NMR studies of Phafin2

**Figure 6:**
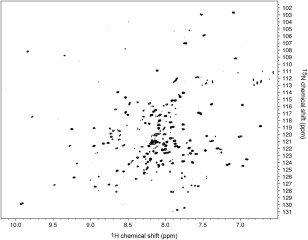
NMR studies of Phafin2. TROSY‐HSQC spectrum of ^1^H, ^15^N‐labeled Phafin2 collected at 30°C.

The structural properties of Phafin2 were further investigated by solution NMR spectroscopy. Figure 6 shows that the ^1^H, ^15^N TROSY‐HSQC spectrum of Phafin2 yielded a single set of resonances, indicating that the protein was highly homogenous at 30°C. In addition, ∼235 HN crosspeaks were identified, which is very close to the expected number (238). The presence of crowded resonances in the center of the ^1^H, ^15^N TROSY‐HSQC spectrum of Phafin2 is consistent with the presence of random coil regions in the protein as deduced from far‐UV CD analysis. NMR spectra of Phafin2 at higher temperatures than 30°C were also recorded, yielding loss of resonances, and aggregation and precipitation of the protein (data not shown).

### Denaturant‐induced unfolding of Phafin2

**Figure 7:**
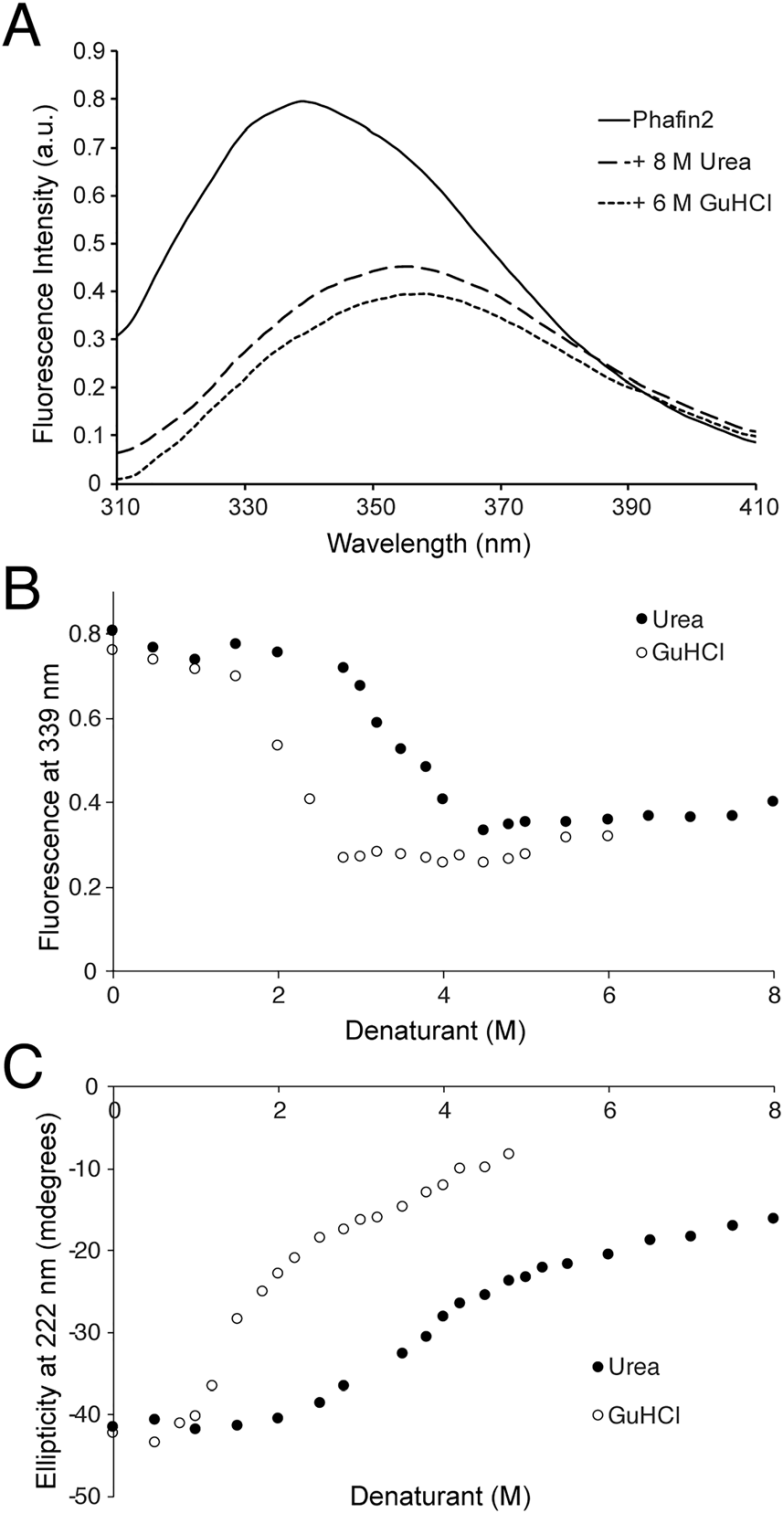
Urea‐ and GuHCl‐mediated denaturation of Phafin2 followed by tryptophan fluorescence and CD spectroscopy. A: Fluorescence emission spectra of Phafin2 in the absence (solid line) and presence of 8*M* urea (dashed line) or 6*M* GuHCl (dotted line). B: Changes in the fluorescence intensity of Phafin2 at 339 nm induced by increasing urea (filled circles) or GuHCl (empty circles) concentrations. C: Changes in the CD ellipticity of Phafin2 at 222 nm induced by increasing urea (filled circles) or GuHCl (empty circles) concentrations.

To study protein folding, the standard Gibbs free energy in water (Δ $G_{H2O}^{0}$) is the most relevant parameter for the quantification of protein stability. Furthermore, the linear extrapolation method is often used for determining Δ $G_{H2O}^{0}$ by the use of chemical denaturants, such as guanidine hydrochloride (GuHCl) and urea.^12^ To monitor denaturant‐induced structural changes of Phafin2, we employed intrinsic tryptophan fluorescence and far‐UV CD studies. Phafin2 emits a fluorescent emission maximum at 339 nm, but it shifts to 356 nm in the presence of 8*M* urea or 6*M* GuHCl [Fig. 7(A)]. By representing the reduction of fluorescence intensity at 339 nm, we found that the unfolding curve of Phafin2 is sigmoidal [Fig. 7(B)]. Thermodynamic analysis showed that the Δ $G_{H2O}^{0}$ was 4.07 and 3.80 kcal mol^−1^ for urea and GuHCl, respectively (Table 2, Supporting Information Fig. S1). Data analysis also indicated that Phafin2 was susceptible to unfolding, showing *C*_m_ (Δ $G_{H2O}^{0}$/m) values of 3.42 and 1.30*M* for urea and GuHCl, respectively (Table 2, Supporting Information Fig. S1). These parameters were also calculated by monitoring structural changes in Phafin2 at 222 nm using CD spectroscopy. The Δ $G_{H2O}^{0}$ and *C*_m_ values of Phafin2 for urea‐induced unfolding were 4 kcal mol^−1^ and 3.45*M*, respectively, whereas GuHCl‐induced unfolding showed Δ $G_{H2O}^{0}$ and *C*_m_ values of 3.75 kcal mol^−1^ and 1.51*M*, respectively (Table 2, Supporting Information Fig. S1). Therefore, the Phafin2 thermodynamic parameters obtained by two independent methodologies were similar. By comparing the urea‐induced unfolding traces obtained by intrinsic tryptophan fluorescence and CD, the Phafin2 structure remained unchanged up to ∼2*M* of urea concentration and reached a maximum denaturation at ∼4.5*M* of the denaturant. In the case of GuHCl‐mediated denaturation, the Phafin2 structure was stable at ∼0.5*M* of GuHCl concentration, but achieved full denaturation at ∼3*M* of the denaturant using both methodologies. Unfolding of small globular proteins occurs through a process that conforms to the two‐state mechanism^12^; however, elongated proteins, such as the Notch ankyrin domain,^13^ spectrin,^14^ and the bacterial surface protein SasG,^15^ among others, exhibit chemical denaturation sigmoidal plots. In this scenario, the population level of intermediate states is insignificant.^16^ The denaturation curves of Phafin2 indicate that the protein follows a two‐state mechanism without the presence of intermediate states. The presence of a single steep transition in these curves suggests that the Phafin2 PH and FYVE domains are thermodynamically coupled, possibly due to interdomain interactions, as observed in other multidomain containing proteins.^17^, ^18^, ^19^

**Table 2:** Thermodynamic Parameters Obtained from Urea‐ and GuHCl‐Induced Denaturation of Phafin2

Condition	Probe	Δ $G_{H2O}^{0}$ (kcal mol^−1^)	*m* (kcal mol^−1^ M^−1^)	*C*_m_ (M)
Urea	Fluorescence	4.07	1.19	3.42
	CD	4.00	1.16	3.45
GuHCl	Fluorescence	3.80	2.93	1.30
	CD	3.75	2.48	1.51

### Analysis of Phafin2 association to PtdIns(3)P

**Figure 8:**
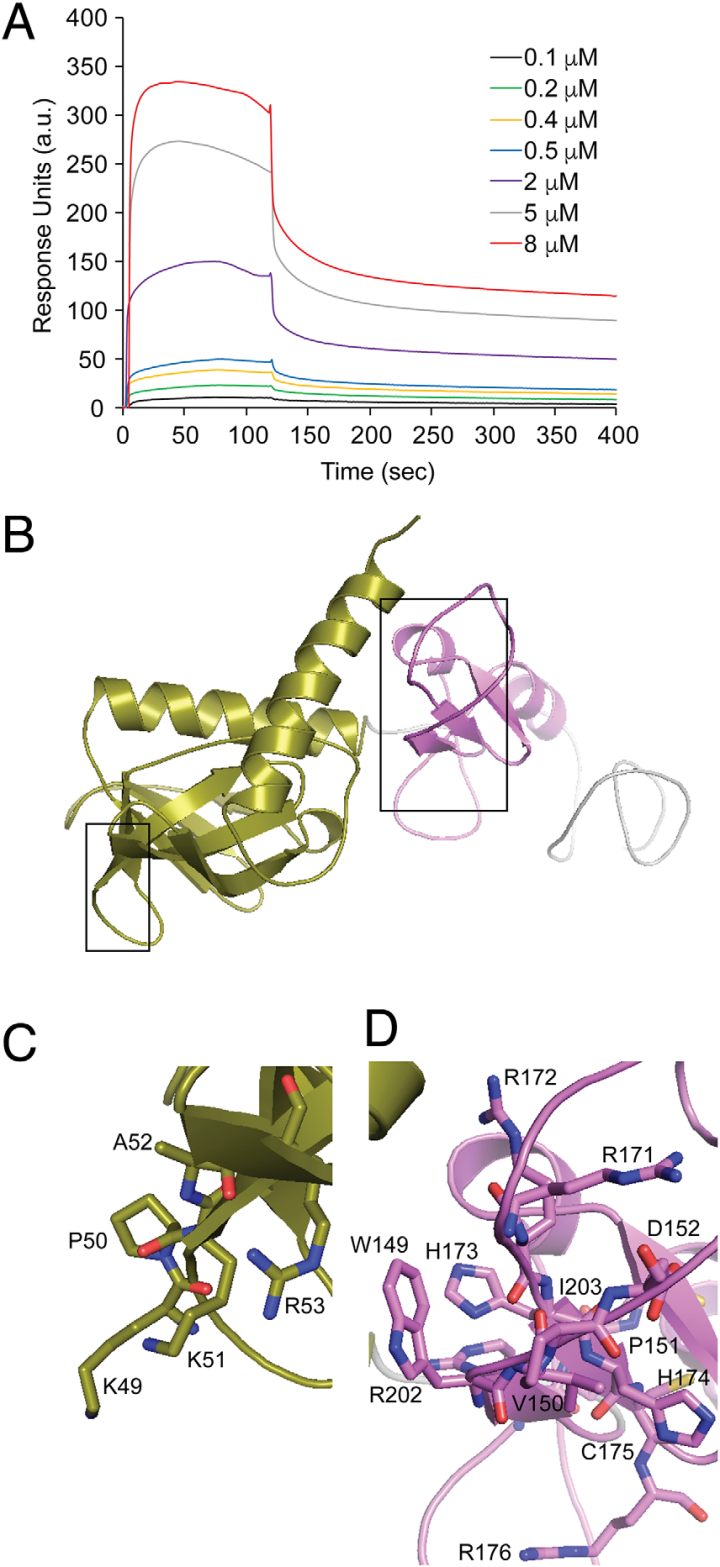
Analysis of Phafin2 binding to PtdIns(3)P. A: Raw SPR sensorgram for the association of Phafin2 to PtdIns(3)P liposomes, in which the lipid was incorporated in liposomes containing dioleoyl phosphatidylcholine and immobilized onto an L1 sensorchip flow cell. The other flow cell contained dioleoyl phosphatidylcholine liposomes employed as a control. Binding kinetics were recorded at room temperature. The concentrations of Phafin2 flown on the sensor chip are color‐coded. a.u., arbitrary units. Data is a representation of four independent experiments. B: Structure of human Phafin2 modeled using Phyre2. The potential PtdIns(3)P‐dependent membrane‐binding sites in the PH domain (left, in olive) and in the FYVE domain (right, in pink) are boxed. C–D: A close‐up view of the putative PtdIns(3)P‐binding sites in the PH (C) and FYVE (D) domains. The regions displayed correspond to the 49‐KPKAR‐53 motif in the PH domain and the 149‐WVPD‐152, 171‐RRHHCR‐176, and 202‐RVC‐204 motifs in the FYVE domain.

To obtain a more quantitative measurement of PtdIns(3)P binding to Phafin2, we performed surface plasmon resonance (SPR) binding analysis using liposomes immobilized on an L1 sensor chip. Measurements were carried out relative to a sensor chip surface containing 100% phosphatidylcholine. Phafin2 bound to PtdIns(3)P liposomes with a fast association rate, but the steady state was transient and response dropped slightly at the end of the injection [Fig. 8(A)]. The interaction best fit the two‐state conformational change model, in agreement with the local conformational changes induced by the phosphoinositide [Fig. 4(B,C)], with an estimated dissociation constant (*K*_D_) of 285 ± 80 n*M* (*χ* ^2^ = 3.5 RU^2^). This value is in agreement with the phosphoinositide affinity values calculated for other PH and FYVE domain‐containing proteins.^20^ PH domains strongly bind to polyphosphoinositides and are mediated, in most cases, by the basic KX_n_(K/R)XR motif,^21^ which is represented by the sequence 49‐KPKAR‐53 sequence in the Phafin2 PH domain. Binding of the PH domain to mono‐phosphate phosphoinositides, such as the case for the Phafin2 PH domain^2^ is rare; a well‐documented example is the GLUE domain of Vps36, which shows a split PH‐domain fold that specifically binds PtdIns(3)P in a mode that differs from other phosphoinositide‐binding domains.^22^ However, FYVE domains bind specifically to PtdIns(3)P with reports showing binding affinities for the lipid ranging from nanomolar to micromolar concentrations.^23^ Lipid and membrane insertion of the FYVE domain is mediated by three consensus sequences, WxxD, RR/KHHCR, and RVC motifs,^20^ all of which are present in the Phafin2 FYVE domain. A model of the structure of human Phafin2 [Fig. 8(B)] was obtained from its protein sequence and homology modeling using Phyre2^24^ with >90% accuracy using the five closest templates (PDB accession codes 3KY9, 1JOC, 3BJI, 2YQM, 2VRW). The elongated predicted structure shows the two putative PtdIns(3)P‐dependent membrane‐binding sites located at predominantly flexible regions [Fig. 8(B–D)]. In addition to PtdIns(3)P, it is possible that the FYVE and PH domains associate to other lipid ligands through acidic electrostatic interactions,^25^ which may be required for Phafin2 targeting to endomembrane compartments.

## Conclusions

In conclusion, we demonstrate that Phafin2 is an α/β moderately elongated and stable monomer. Unfolding traces that were measured using urea and GuHCl indicate that Phafin2 denatures in a two‐state transition model. The lack of an intermediate state suggests that the PH and FYVE domains cooperate during the unfolding process. Phafin2 interacts with PtdIns(3)P with nanomolar affinity, inducing local conformational changes in its tertiary structure. The PtdIns(3)P binding sites are predicted to be located in flexible regions in the protein. The quality of the purified and stable Phafin2 at room temperature makes the protein appropriate for solution NMR structural studies, which can contribute to a better understanding of its molecular interactions in PtdIns(3)P‐enriched endomembrane compartments.

## Materials and Methods

### Protein expression and purification

The Phafin2 cDNA was isolated from a human liver cDNA library (Clontech) by PCR and cloned into a pGEX4T3 vector (GE Healthcare). Protein was expressed in *Escherichia coli* (Rosetta; Stratagene). Bacterial cells were grown in Luria‐Bertani media at 37°C until cells reached an optical density of ∼0.8. Induction of the GST fusion Phafin2 resulted from the addition of 1 m*M* isopropyl β‐d−1‐thiogalactopyranoside and 1 μ*M* ZnCl_2_ followed by 4‐h incubation at 25°C. Phafin2 was purified using the glutathione bead‐based procedure as previously described.^26^ Proteins were further purified using an FPLC‐driven Superdex 75 column (GE Healthcare) previously equilibrated with 50 m*M* Tris‐HCl (pH 8) and 0.5*M* NaCl. Fractions containing the purified protein were pooled and concentrated in the indicated buffers for biochemical or biophysical analysis. Protein concentration was calculated using the UV‐light absorbance method at 280 nm.

### Mass spectrometry and N‐terminal sequencing analyses

Phafin2 (50 m*M* Tris‐HCl, pH = 8) was reduced with 1,4‐dithiothreitol (5 m*M*) in the presence of urea (8*M*), and digested with trypsin at a substrate‐enzyme ratio of 50:1 w/w (37°C, overnight). After cleanup with a C18 cartridge, a solution of Phafin2 (0.5 µ*M*, 8 µL) was injected in a micro HPLC system (HPLC 1100, Agilent Technologies) and delivered for MS analysis at ∼180 nL/min with a 4 h long gradient of 10% to 100% solvent B (where solvent A was H_2_O:CH_3_CN:TFA, 98/2/0.01 v/v; and, solvent B was H_2_O:CH_3_CN:TFA, 10/90/0.01 v/v). The HPLC system was adapted in‐house to operate in the nano flow mode. MS analysis was performed with a linear trap quadruple MS system (Thermo Electron, San Jose, CA) with the ESI voltage set at +2.0 kV. Tandem MS data were acquired using data dependent analysis on the top five most intense peaks using parameters described in Ref. 27. The database search was performed with Proteome Discoverer 1.4 (Thermo Electron) against an *E. coli* database appended with the sequence of Phafin2. The *E. coli* database was used to enable the identification of possible contaminants from the expression system of Phafin2. Post‐translational modifications were not allowed in the search, the allowed missed cleavage sites was set to 2, and the false discovery rate was set at <3%.^28^ Using these experimental conditions, Phafin2 (Q9H8W4) could be identified by up to 29 unique peptides accounting for a sequence coverage over 70%. Intact Phafin2 was also subjected to N‐terminal sequencing at the Tufts University Core Facility (Boston, MA).

### Sedimentation velocity AUC

Sedimentation velocity AUC was carried out at the Center for AUC of Macromolecular Assemblies at the University of Texas Health Science Center, San Antonio using a Beckman Optima XL‐I analytical ultracentrifuge with absorbance and interference optical detection systems (Beckman Coulter). Sedimentation velocities were analyzed using the UltraScan3 software suite as described (Ref. 29, http://www.ultrascan.uthscsa.edu). Phafin2 (10 μΜ) was prepared in a buffer containing 8 m*M* Tris‐HCl (pH = 7.3) and 50 m*M* NaCl. Absorbance data was obtained at a wavelength of 230 nm at 20°C, and at a rotor speed of 50,000 rpm using standard double‐channel centerpieces. Data were first subjected to 2D spectrum analysis with simultaneous removal of time‐invariant noise^30^ followed by a parametrically constrained spectrum analysis ‐ Monte Carlo analysis.^31^

### Dynamic light scattering

DLS experiments were performed at 25°C using a Malvern Zetasizer Nano‐ZS instrument. DLS studies were with Phafin2 (0.8 mg/mL) in 5 m*M* sodium citrate (pH = 7.3) and 50 m*M* KF. Each run was recorded for 120 s and three accumulated runs were averaged with protein samples previously equilibrated for 2 min.

### CD spectroscopy

Far‐UV CD spectra were obtained using Phafin2 (20 μ*M*) in 5 m*M* sodium citrate (pH = 7.3) and 50 m*M* KF on a Jasco J‐815 spectropolarimeter. Spectra were collected in a 1‐mm path length quartz cell at 25°C. Three accumulated scans of the protein from 250 to 190 nm were recorded using a bandwidth of 1‐nm and a response time of 1 s at a scan speed of 20 nm/min. Secondary structure content of Phafin2 was estimated using the CDSSTR algorithm available at Dichroweb (http://dichroweb.cryst.bbk.ac.uk/html/process.shtml). Three accumulated near‐UV CD spectra of Phafin2 (40 μ*M*) were collected using a 1‐mm path length at 20 nm/min between 350 and 250 nm with a response time of 1 s and a data pitch of 0.5 nm. All CD spectra were corrected for buffer background. Far‐UV CD signal changes at 222 nm were monitored as a function of increasing temperature from 20 to 90°C, with steps of 1°C and with an equilibration time of 30 s at each temperature before recording a reading. For the urea‐ and GuHCl‐induced unfolding experiments, Phafin2 was incubated for 1 h at room temperature with the indicated concentrations of the denaturant before the spectra were recorded.

### Tryptophan fluorescence

Intrinsic tryptophan fluorescence emission spectra of Phafin2 (0.25 μ*M*), in 5 m*M* sodium citrate (pH 7.3) and 50 m*M* KF, were recorded after excitation of the protein at 295 nm on a Jasco J‐815 spectropolarimeter. Emission spectra were collected between 300 and 400 nm using a 10‐mm quartz cuvette at room temperature. Phafin2 was titrated with increasing concentrations of PtdIns(3)P (0.25–16 μ*M*). For the urea‐ and GuHCl‐induced unfolding experiments, Phafin2 was incubated for 1 h at room temperature with the indicated concentrations of the denaturant before the spectra were recorded.

### NMR spectroscopy

NMR experiments of ^1^H, ^15^N Phafin2 (200 μ*M*) in 20 m*M d*_11_Tris‐HCl (pH 7.3), 100 m*M* NaCl, 2 m*M d*_18_ DTT, 1 m*M* NaN_3_, 10% D_2_O were performed on a Bruker AVANCE III 800 MHz NMR spectrometer equipped with a cryoprobe (University of Virginia). Two dimensional [^15^N,^1^H]‐transverse relaxation optimized spectroscopy (TROSY)‐heteronuclear single quantum coherence (HSQC) was performed for the protein sample. Data were processed and analyzed using Topspin 3.2 and NMRpipe.^32^

### Analysis of denaturant unfolding transitions

The transition curves acquired after representing each spectral property (i.e., fluorescence changes of Phafin2 from *F*_339_ to *F*_356_, CD spectral changes of Phafin2 at 222 nm) against denaturant concentration were analyzed to calculate the Gibbs free energy of unfolding (Δ $G_{H2O}^{0}$) in the absence of denaturant. To calculate Δ $G_{H2O}^{0}$ for each spectral analysis, a model in which monomeric native Phafin2 (*f*_N_) was converted to a denatured (*f*_D_) form without the presence of highly populated intermediates was first considered. This state, known as the equilibrium two‐state is represented as:

(1) $$K_{\text{eq}}=\;f_{D}/f_{N}$$

The estimation of *K*_eq_ was used to calculate the dependence of the standard Gibbs energy of denaturation for each denaturant concentration (Δ*G*) using the relation:

(2) $$\Delta G=\;-\mathrm{RT}\;\text{ln}\;K_{\text{eq}}=\;-\mathrm{RT}\;\text{ln}\;\left(f_{D}/1-f_{D}\right)$$

where *R* is the universal gas constant (1.986 cal mol^−1 ^K^−1^) and *T* is the temperature on the Kelvin scale. The Δ*G* value varies linearly with denaturant concentration following the equation:

(3) $$\Delta G\;\left(x\right)\;=\;\Delta G^{0}{}_{H2O}+m\left[x\right]$$

where [*x*] is the molar concentration of the denaturant and *m* is the slope of the plot. Thus, linear plots of Δ*G* versus denaturant concentration were obtained. Data was fitted to the nonlinear least‐squares method using Microsoft Excel (Microsoft Corporation, Redmond WA) for the calculation of the Δ $G_{H2O}^{0}$ and *m* for each experimental condition.^12^ Using these parameters, the *C*_m_, the denaturant concentration at the midpoint of the unfolding transition when Δ*G* is 0, was estimated. Similarly, the *T*_M_ of Phafin2 was calculated by following the denaturation of the protein by its CD ellipticity at 222 nm and by determining the temperature at which Δ*G* is 0 using Eq. (2).

### SPR analysis

Liposomes were prepared as previously described.^33^ Briefly, lipids including 1‐palmitoyl‐2‐oleoyl‐*sn*‐glycero‐3‐phosphatidylcholine (DOPC; control), or DOPC and dipalmitoyl PtdIns(3)P (1.5%) were dissolved in chloroform/methanol/water (65:35:0.8). The lipid mixture was first dried under N_2_ and further under vacuum to remove residual chloroform. Phospholipids were resuspended in 20 m*M* HEPES (pH 7.0) and 100 m*M* NaCl to a final concentration of 4 m*M*, sonicated, and extruded for 100‐nm liposome size at 65°C. SPR analysis was performed on a BIAcore X100 instrument with a liposome‐coated L1 sensor chip at room temperature. Typical liposome loading was ∼5,000 RU/channel. Kinetic SPR measurements were performed with the flow rate set at 30 μL/min. Apparent *K*_D_ values were estimated using the BIAevaluation software, version 2.0 (GE Healthcare). Experiments determining *K*_D_ values for PtdIns(3)P were carried out by collecting four independent experiments.

## Supporting information

Supporting Information
