# Supplementary material for: Structural, thermodynamic, and phosphatidylinositol 3‐phosphate binding properties of Phafin2
Source: Protein Sci. 2017 Feb 13;26(4):814–23. doi: 10.1002/pro.3128 (PMC5368057; doi:10.1002/pro.3128)
Supplement: Supplementary file 1 — Supporting Information [file PRO-26-814-s001.docx]

**Supplementary Information for**

**Structural, thermodynamic, and phosphatidylinositol 3-phosphate**

**binding properties of Phafin2**

Tuo-Xian Tang, Ami Jo, Jingren Deng, Jeffrey F. Ellena,

Iulia M. Lazar, Richey M. Davis, and Daniel G. S. Capelluto ^*^

**Table S1.** Identification of human Phafin2. MS/MS results obtained on additional 16 peptides generated by trypsin proteolysis of Phafin2.

| Peptide | Phafin2 | Sequence |
| --- | --- | --- |
| 1 | 15-34 | ISIVENCFGAAGQPLTIPGR |
| 2 | 35-44 | VLIGEGVLTK |
| 3 | 74-91 | YNKQHIIPLENVTIDSIK |
| 4 | 77-91 | QHIIPLENVTIDSIK |
| 5 | 77-97 | QHIIPLENVTIDSIKDEGDLR |
| 6 | 77-103 | QHIIPLENVTIDSIKDEGDLRNGWLIK |
| 7 | 104-119 | TPTKSFAVYAATATEK |
| 8 | 107-128 | SFAVYAATATEKSEWMNHINK |
| 9 | 137-160 | SGKTPSNEHAAVWVPDSEATVCMR |
| 10 | 140-160 | TPSNEHAAVWVPDSEATVCMR |
| 11 | 140-163 | TPSNEHAAVWVPDSEATVCMRCQK |
| 12 | 177-190 | KCGFVVCGPCSEK |
| 13 | 178-190 | CGFVVCGPCSEK |
| 14 | 192-202 | FLLPSQSSKPVR |
| 15 | 203-223 | ICDFCYDLLSAGDMATCQPAR |
| 16 | 233-249 | SPLNDMSDDDDDDDSSD |
|  |  |  |


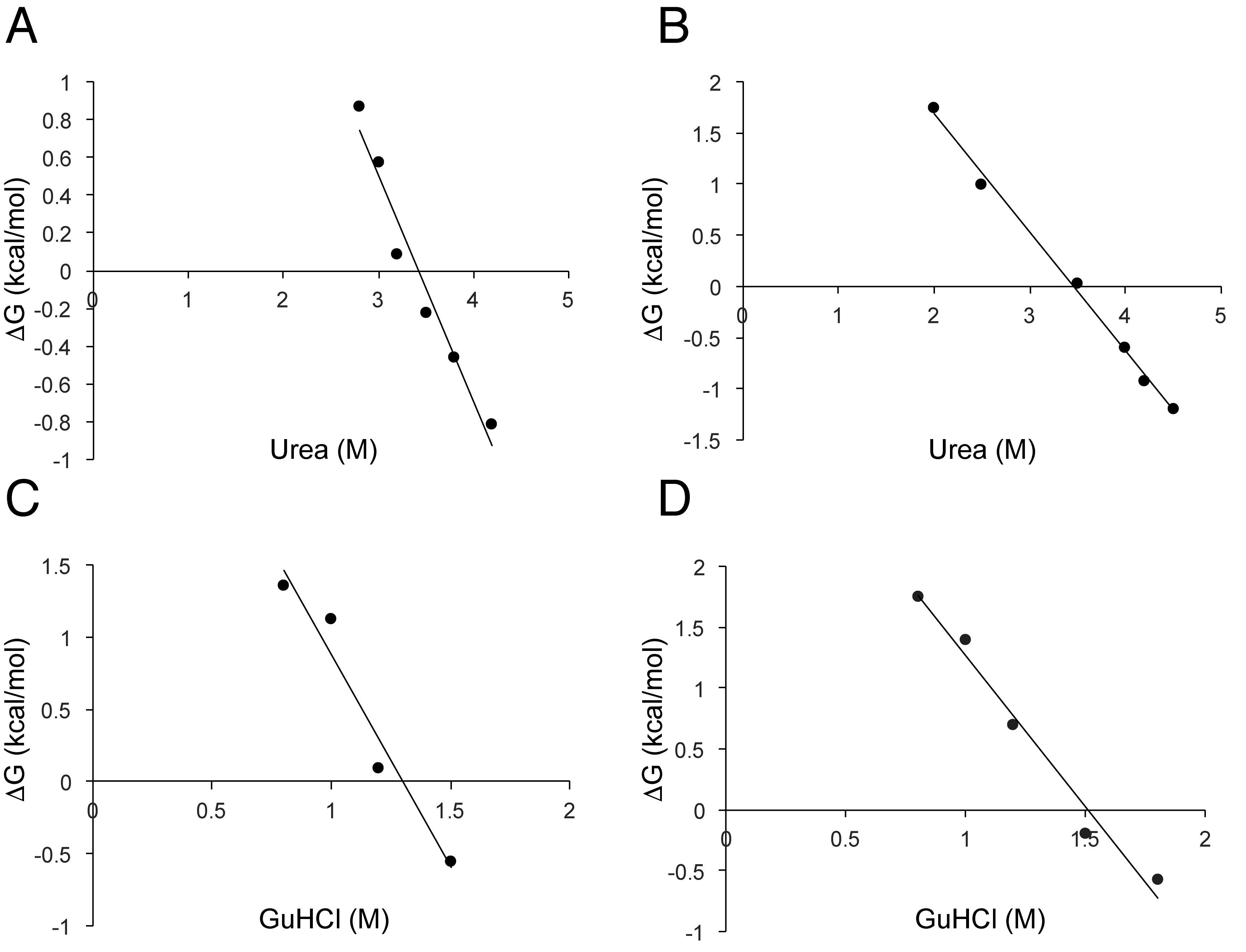


**Supplementary Figure 1.** Gibbs free energy change (ΔG) in the transition region plotted as a function of the denaturant concentration. (A-B) Urea denaturation plots of Phafin2 monitored by intrinsic tryptophan fluorescence (A) and far-UV circular dichroism (B). (C-D) Guanidine hydrochloride denaturation plots of Phafin2 monitored by intrinsic tryptophan fluorescence (C) and far-UV circular dichroism (D). The ΔG^0^_H2O_ was estimated from the intercept on the Y axis using the linear extrapolation method.
